# Response of Soil C and N, Dissolved Organic C and N, and Inorganic N to Short-Term Experimental Warming in an Alpine Meadow on the Tibetan Plateau

**DOI:** 10.1155/2014/152576

**Published:** 2014-05-22

**Authors:** Cheng-Qun Yu, Zhen-Xi Shen, Xian-Zhou Zhang, Wei Sun, Gang Fu

**Affiliations:** Lhasa Plateau Ecosystem Research Station, Key Laboratory of Ecosystem Network Observation and Modeling, Institute of Geographic Sciences and Natural Resources Research, Chinese Academy of Sciences, Beijing 100101, China

## Abstract

Although alpine meadows of Tibet are expected to be strongly affected by climatic warming, it remains unclear how soil organic C (SOC), total N (TN), ammonium N (NH_4_^+^-N) , nitrate N (NO_3_^+^-N), and dissolved organic C (DOC) and N (DON) respond to warming. This study aims to investigate the responses of these C and N pools to short-term experimental warming in an alpine meadow of Tibet. A warming experiment using open top chambers was conducted in an alpine meadow at three elevations (i.e., a low (4313 m), mid-(4513 m), and high (4693 m) elevation) in May 2010. Topsoil (0–20 cm depth) samples were collected in July–September 2011. Experimental warming increased soil temperature by ~1–1.4°C but decreased soil moisture by ~0.04 m^3^ m^−3^. Experimental warming had little effects on SOC, TN, DOC, and DON, which may be related to lower warming magnitude, the short period of warming treatment, and experimental warming-induced soil drying by decreasing soil microbial activity. Experimental warming decreased significantly inorganic N at the two lower elevations,but had negligible effect at the high elevation. Our findings suggested that the effects of short-term experimental warming on SOC, TN and dissolved organic matter were insignificant, only affecting inorganic forms.

## 1. Introduction

Soil organic C (SOC) and total N (TN) are very important C and N pools in the terrestrial ecosystems [1, 2]. As the components of labile C and N pools in soils, dissolved organic C (DOC) and N (DON) and soil ammonium and nitrate N (NH_4_^+^-N and NO_3_^−^-N) play crucial roles in the biogeochemistry of C and N and in the nutrient transformation [3–5]. With the context of climatic warming, how SOC, TN, DOC, DON, NH_4_^+^-N, and NO_3_^−^-N respond is vital to global C and N cycling [1, 2]. However, inconsistent results on the responses of these C and N pools to climatic warming have been observed with respect to vegetation types and initial soil characteristics [2, 3, 6–14]. For example, He et al. [2] demonstrated that six-year warming (~1.4°C increase of 10 cm soil temperature) significantly decreased soil C by 129.3 g m^−2^ in a temperate steppe of Inner Mongolia. In contrast, Li et al. [7] found that two-year warming significantly increased SOC in an alpine meadow (~2.1°C increase of air temperature) but significantly reduced TN in an alpine swamp meadow (~2.3°C increase of air temperature) on the Tibetan Plateau. Hagedorn et al. [13] indicated that one-growing-season warming (~4°C increase of 5 cm soil temperature) did not significantly influence DOC. Song et al. [1] pointed out that six-year warming (~1.2°C increase of 10 cm soil temperature) significantly reduced DOC in a temperate steppe in Inner Mongolia. Biasi et al. [15] indicated that two-year warming (~0.9°C increase of 5 cm soil temperature) did not have obvious effects on DON, NH_4_^+^-N, NO_3_^−^-N, and N_min⁡_ in a lichen-rich dwarf shrub tundra in Siberia. Bai et al. [14] stated that experimental warming (~0.6–6.7°C in soil temperature) had a significant positive effect on N_min⁡_ but not on TN across all biomes. Therefore, how climatic warming acts on C and N cycling still remains unclear. 

More than 70% of the Tibetan Plateau is covered with grasslands [16]. The alpine grasslands of this Plateau are one of the systems most sensitive to global change [17, 18]. In alpine grasslands, understanding the responses of SOC, DOC, TN, DON, NH_4_^+^-N, and NO_3_^−^-N to climatic warming are crucial for predicting future changes in soil fertility and C sequestration. The alpine meadow is one of the most typical grasslands types on the Tibetan Plateau being subjected to climatic warming [19]. Information on how these C and N pools along an elevation gradient respond to climatic warming is scarce on the Tibetan Plateau. Here we set up a warming experiment in an alpine meadow at three elevations (i.e., 4313 m, 4513 m, and 4693 m) on the Northern Tibetan Plateau.

The main objective was to investigate the effects of short-term experimental warming on SOC, TN, DOC, DON, NH_4_^+^-N, and NO_3_^−^-N. Our previous study indicated that short-term experimental warming could not affect soil microbial biomass [20] and soil microbial activity regulated the balances of soil C and N pools in the alpine meadow [21]. We hypothesized that experimental warming may not affect these C and N pools in this study.

## 2. Materials and Methods

### 2.1. Study Area, Experimental Design, and Soil Sampling

A detailed description of the study area, the warming experimental design, the measurements of microclimate factors (including soil temperature and soil moisture), and the soil sampling are given in Fu et al. [20, 22].

Briefly, three alpine meadow sites were established at three elevations (i.e., a low (30°30′N, 91°04′E, and 4313 m), mid- (30°31′N, 91°04′E, and 4513 m), and high (30°32′N, 91°03′E, and 4693 m) elevation) at Damxung Grassland Observation Station of Tibet Autonomous Region in China in May 2010.

Annual mean air temperature and precipitation is 1.3°C and ~476.8 mm, respectively [20, 21]. The vegetation is* Kobresia*-dominated alpine meadow and roots are mainly concentrated in the topsoil layer (0–20 cm) [21, 22]. The soil is classified as sandy loam, with pH of 6.0–6.7, organic matter of 0.3–11.2%, and total N of 0.03–0.49% [20, 22].

Open top chambers (OTCs, 3 mm thick polycarbonate) were used to enhance temperature [22, 23]. The bottom and top diameters and the height of OTCs were 1.45 m and 1.00 m and 0.40 m, respectively [20, 22]. For each site, four OTCs and their paired control plots (1 m × 1 m) were randomly established in May 2010. There was ~3 m distance between plots.

Daily mean soil temperature (*T*_*s*_) during the study period of July-September in 2011 inside the OTCs increased by 1.26°C, 0.98°C, and 1.37°C at the low, mid-, and high elevation, respectively, compared to control plots [20]. In contrast, experimental warming decreased daily mean soil moisture (SM) by 0.04 m^3^ m^−3^ in all sites [20]. Daily mean *T*_*s*_ decreased with increasing elevation from the low to high elevation [20].

We collected topsoil samples (0–20 cm depth) inside each plot using a probe 3.0 cm in diameter on July 7, August 9, and September 10, 2011 [20]. Five soil subsamples were randomly sampled and composited into one soil sample for each plot [20]. Subsamples of the fresh soil were used to measure DOC, DON, NH_4_^+^-N, and NO_3_^−^-N and other subsamples of the fresh soil were air-dried for the measurements of SOC and TN.

### 2.2. Soil Analysis

A more detailed description of measurements of soil inorganic N (N_min⁡_, i.e., sum of NH_4_^+^-N and NO_3_^−^-N), DON, and DOC can be found in Fu et al. [21]. Briefly, soil inorganic N in 20 g fresh soil sample was extracted with 100 mL K_2_SO_4_, filtered through 0.45 *μ*m membrane, and analyzed on a LACHAT Quikchem Automated Ion Analyzer. Dissolved organic C and TN (DTN) in another 20 g fresh soil sample was extracted with 100 mL ultrapure water and filtered through 0.45 membrane. The extractable SOC and TN concentrations in the ultrapure water extracts were measured using a Liqui TOC II elementar analyzer (Elementar Liqui TOC, Elementar Co., Hanau, Germany) and a UV-1700 PharmaSpec visible spectrophotometer (220 nm and 275 nm), respectively. We also analyzed dissolved inorganic N (DIN) in the ultrapure water extracts on a LACHAT Quikchem Automated Ion Analyzer. Then DON was calculated as the difference between DTN and DIN. The potassium dichromate method was used to determine SOC [24]. Soil TN was measured on a CN analyzer (Elementar Variomax CN). Soil microbial biomass (MBC) and N (MBN) data were obtained from Fu et al. [20].

### 2.3. Statistical Analysis

In order to examine the elevation effect, repeated-measures ANOVA with experimental warming and elevation as the between subject factors and with sampling date as the within subject factor was performed for a specific soil property (i.e., SOC, TN, DOC, DON, ratio of DOC to DON (DOC/DON), NH_4_^+^-N, NO_3_^−^-N, ratio of NH_4_^+^-N to NO_3_^−^-N(NH_4_^+^-N/NO_3_^−^-N), and N_min⁡_). At each site, repeated-measures ANOVA with experimental warming (i.e., OTCs versus control) as the between subject factor and with sampling date as the within subject factor was conducted for each soil property. Single factor linear regressions were performed between soil properties and *T*_*s*_, SM, MBC, and MBN. In addition, multiple stepwise regression analyses were conducted for soil properties to examine the relative importance of *T*_*s*_, SM, MBC, and MBN in affecting the variations of soil properties. All data were examined for normality and homogeneity before analysis and natural logarithm transformations were made if necessary. The level of significance was *P* < 0.05. All the statistical tests were performed using the SPSS software (version 16.0; SPSS Inc., Chicago, IL).

## 3. Results

### 3.1. Effects of Experimental Warming on Soil Properties

**Figure 1:**
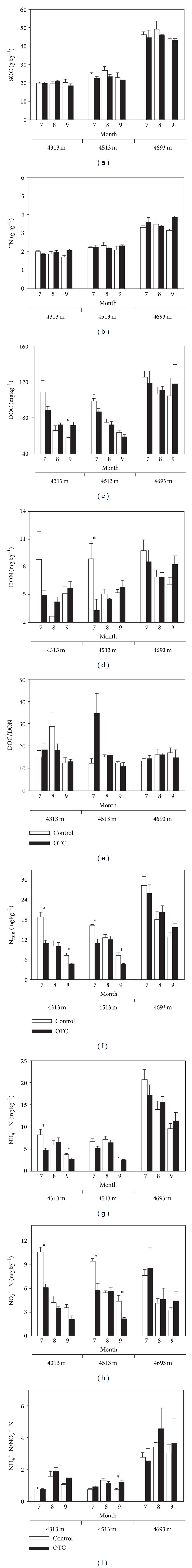
Effects of experimental warming on soil organic C (SOC), total N (TN), dissolved organic C (DOC), dissolved organic N (DON), the ratio of DOC to DON (DOC/DON), soil inorganic N (N_min⁡_), ammonium N (NH_4_^+^-N), nitrate N (NO_3_^−^-N), and the ratio of NH_4_^+^-N to NO_3_^−^-N(NH_4_^+^-N/NO_3_^−^-N) in the three alpine meadow sites located at elevation 4313 m, 4513 m, and 4693 m, respectively (mean ± SE, *n* = 4). *indicates *P* < 0.05, while no asterisk indicates not significant.

Regardless of experimental warming, elevation had significant effects on SOC (*F* = 183.19, *P* < 0.001), TN (*F* = 126.38, *P* < 0.001), DOC (*F* = 26.42, *P* < 0.001), DON (*F* = 7.08, *P* < 0.01), NH_4_^+^-N(*F* = 71.98, *P* < 0.001), NH_4_^+^-N/NO_3_^−^-N(*F* = 14.01, *P* < 0.001), and N_min⁡_(*F* = 56.29, *P* < 0.001) across the three sampling dates. In contrast, there were no significant effects of elevation on NO_3_^−^-N and DOC/DON. These C and N pools showed similar seasonal dynamics regardless of experimental warming among the three elevations (Figure 1).

In line with our initial hypothesis, experimental warming had little effects on SOC, TN, DOC, DON, DOC/DON, and NH_4_^+^-N/NO_3_^−^-N (Table 1). In contrast, the sensitivity of N_min⁡_ to experimental warming increased with increasing elevation (Table 1). In detail, experimental warming significantly decreased N_min⁡_ by 29.2% and 23.5% at the low and mid-elevation, NO_3_^−^-N by 36.4%, 29.5% at the low and mid-elevation, and NH_4_^+^-N by 16.7% at the mid-elevation across all the three sampling dates, respectively. In contrast, experimental warming had little effects on NO_3_^−^-N and N_min⁡_ at the high elevation.

### 3.2. Relationships between Soil Properties and Environmental Variables and Soil Microbial Biomass

**Figure 2:**
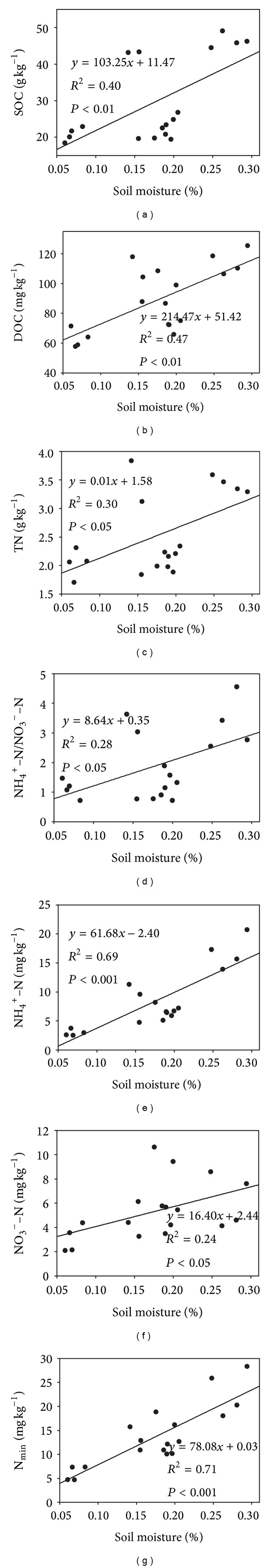
Relationships of soil moisture with soil organic C (SOC), dissolved organic C (DOC), total N (TN), the ratio of NH_4_^+^-N to NO_3_^−^-N(NH_4_^+^-N/NO_3_^−^-N), ammonium N (NH_4_^+^-N), nitrate N (NO_3_^−^-N), and soil inorganic N (N_min⁡_).

Soil organic C, TN, DOC, NH_4_^+^-N, NO_3_^−^-N, NH_4_^+^-N/NO_3_^−^-N, and N_min⁡_ were significantly and positively correlated with SM (Figure 2). In contrast, SOC, TN, DOC, NH_4_^+^-N, and NH_4_^+^-N/NO_3_^−^-N declined with increasing *T*_*s*_ (Table 2). The negative correlations of *T*_*s*_ with DON and N_min⁡_ were relatively lower (Table 2). Soil organic C, TN, DOC, DON, NH_4_^+^-N, NH_4_^+^-N/NO_3_^−^-N, and N_min⁡_ increased significantly with increasing MBC and MBN, while NO_3_^−^–N only increased significantly with increasing MBN (Table 2). Nitrate N was not related to MBC and *T*_*s*_ (Table 2), while DON was not correlated with SM (data not shown). In addition, DOC/DON was not correlated with *T*_*s*_, SM, MBC, and MBN (data not shown).

The multiple stepwise regression analyses were listed in Table 3. Both SOC and TN were simultaneously affected by MBC and *T*_*s*_, whereas MBC explained more variation of the two soil properties than *T*_*s*_. Only MBC was included in the multiple regression equations for DOC, DON, and NH_4_^+^-N/NO_3_^−^-N, while only MBN was included in the regression equation for NO_3_^−^-N. Soil microbial biomass C explained the variation of NH_4_^+^-N more than SM. Both MBC and MBN were simultaneously and positively correlated with N_min⁡_. In addition, all the five concerned variables were excluded for DOC/DON.

## 4. Discussion

### 4.1. Effects of Experimental Warming on SOC, TN, DOC, and DON

Recently, some studies showed that short-term (<3 years) experimental warming had little effects on SOC, TN, DOC, and/or DON in a tallgrass prairie with a silt loam soil (~2°C increase of 5 cm soil temperature) in USA [25], in a dragon spruce plantation with a mountain brown soil (~0.6°C increase of 5 cm soil temperature) on the Tibetan Plateau [8], in an alpine treeline with a sandy Ranker and Podzols soil (~4°C increase of 5 cm soil temperature) in Switzerland [13], and in a lichen-rich dwarf shrub tundra with Gleyic Cryosols soils (~0.9°C increase of 5 cm soil temperature) in Siberia [15]. However, other studies with long-term (>3 years) experimental warming indicated that warming significantly increased or decreased SOC, TN, DOC, and/or DON in a temperate steppe with a Calcic Kastanozems soil in Inner Mongolia (~1.4°C increase of 10 cm soil temperature) [2], in an alpine meadow (~3°C increase of 5 cm soil temperature) on the Tibetan Plateau [3], and in a temperate steppe with chestnut soil in Inner Mongolia (~1.2°C increase of 10 cm soil temperature) [1]. Therefore, the insignificant responses of SOC, TN, DOC, and DON to warming (Table 1) may be due to the short period of warming treatment (14–16 months).

A meta-analysis showed that the effects of experimental warming on N_min⁡_, net N mineralization, and nitrification were significantly and positively correlated with raised soil temperature (~0.6–6.7°C for N_min⁡_, ~0.6–5.5°C for net mineralization, and ~1.3–5.5°C for net nitrification) across all biomes [14]. Similarly, we found that experimental warming-induced change of soil temperature tended to be negatively correlated with that of TN (*R* ^2^ = 0.43, *P* = 0.057) and positively correlated with that of MBN (*R* ^2^ = 0.43, *P* = 0.056) [20]. In addition, MBN was significantly correlated with SOC, TN, DOC, and DON (Table 2). Therefore, the negligible responses of soil C and N pools to experimental warming (Table 1) may be also due to lower warming magnitude in this alpine meadow.

Microbial activity regulates the production of dissolved organic matter [5, 8, 26] and experimental warming-induced decline in soil moisture may suppress soil microbial activity [20, 27]. Similarly, we also found that soil C and N pools increased with increasing soil microbial biomass and soil moisture (Figure 2, Table 2). Moreover, short-term experimental warming had little effect on soil microbial biomass in this system [20]. Therefore, the negligible responses of SOC, TN, DOC, and DON to short-term experimental warming may be also related to that of soil microbial biomass [8, 20]. Moreover, experimental warming-induced soil drying may also suppress the production of DOC and DON [8, 20].

### 4.2. Effects of Experimental Warming on Soil Inorganic N

Bai et al. [14] demonstrated that experimental warming did not significantly increase net N nitrification in grasslands. Similarly, experimental warming did not increase net N mineralization in an alpine meadow on the Tibetan Plateau [28]. In the same alpine meadow as this study, the finding that experimental warming did not increase ecosystem photosynthesis and aboveground plant biomass [22] also indirectly supported that experimental warming may not increase soil N availability because it has been observed that plant productivity is positively correlated with net N mineralization [29]. Therefore, the negligible or negative effect of experimental warming on soil inorganic N (Figure 1, Table 1) may result from the suppression of net N mineralization and nitrification under warming.

The suppression of net N mineralization and nitrification may be owing to decreases in soil moisture and microbial activity because N_min⁡_, NH_4_^+^-N, and NO_3_^−^-N increased significantly with increasing soil moisture and microbial biomass (Figure 2, Table 2). Similarly, the experimental warming-induced significant reductions or insignificant changes of inorganic N (Figure 1, Table 1) were also partly attributed to experimental warming-induced decline in soil microbial biomass [20] and soil drying [10, 29, 30]. This was in line with the finding that the effect of experimental warming on soil moisture was significantly correlated with that on soil nitrification [14]. On the other hand, microbial biomass was more closely related to soil inorganic N than soil moisture (Table 3). This implied that microbial biomass may dominate the variation of soil inorganic N in this study. However, our previous study showed that short-term experimental warming tended to reduce microbial biomass due to soil drying in the same alpine meadow as this study [20]. Therefore, the experimental warming-induced changes of soil inorganic N, net N mineralization, and nitrification may be directly related to that of microbial activity and indirectly related to that of soil moisture.

**Figure 3:**
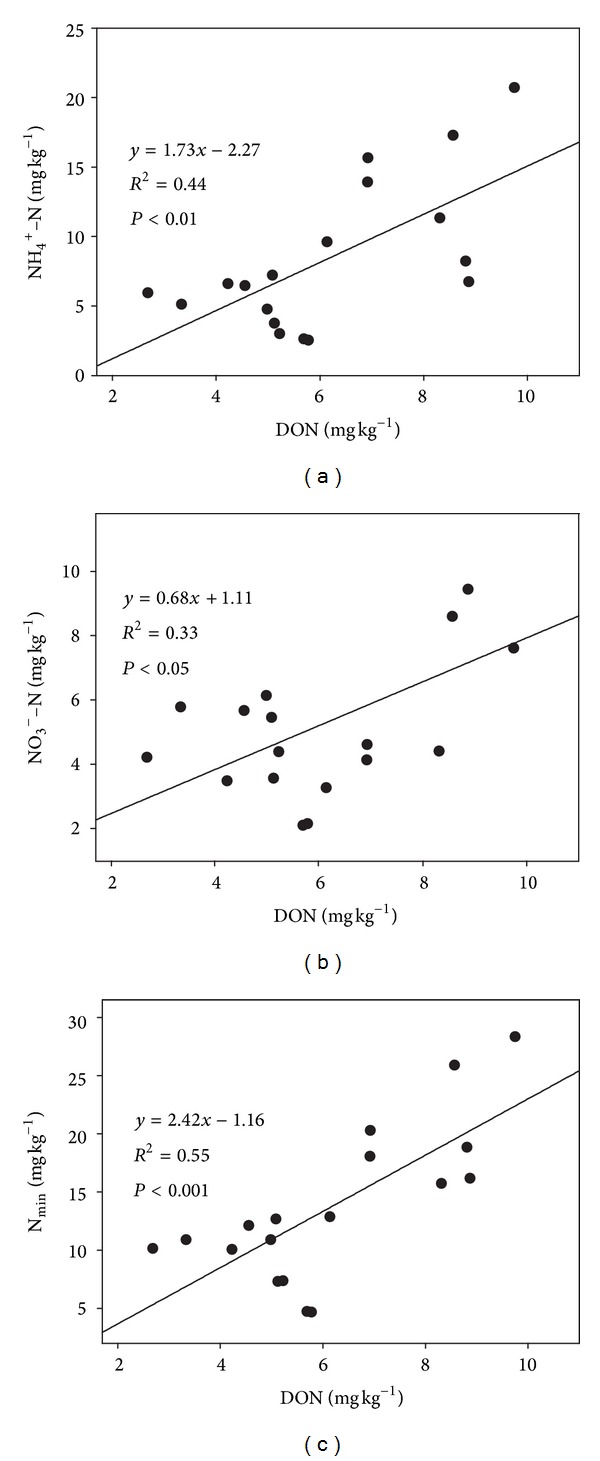
Relationships of dissolved organic N (DON) with ammonium N (NH_4_^+^-N), nitrate N (NO_3_^−^-N), and soil inorganic N (N_min⁡_).

The different responses of N_min⁡_ to experimental warming among the three elevations across the sampling dates could be attributed to several probable underlying mechanisms. First, DON is high-quality N source for N mineralization [8, 31]. This was supported by the positive relationships between DON and N_min⁡_ and NH_4_^+^-N and NO_3_^−^-N (Figure 3). DON under warmed plots tended to be decreased by 10.3% at the low elevation and by 28.7% at the mid-elevation but to be increased by 4.4% at the high elevation across all the three sampling dates, compared to control plots. Second, experimental warming-induced different changes in soil microbial biomass N (MBN) among three elevations [20] could partly explain this phenomenon considering that the production of DON and the immobilization of soil inorganic N were regulated by MBN [3, 32, 33]. This viewpoint was confirmed by the positive correlations between MBN and DON, N_min⁡_, NH_4_^+^-N, and NO_3_^−^-N (Table 2). Third, the response of soil N availability to warming could be strongly related to the initial conditions [8, 34]. In our system, N_min⁡_, DON, and microbial biomass at the high elevation were significantly larger compared to the low and mid-elevation, whilst there were insignificant differences between the latter two [20].

## 5. Conclusions

In summary, short-term experimental warming had no obvious effects on topsoil organic C, total N, dissolved organic C, and N pools for the alpine meadow in this study. The insignificant responses of these C and N pools to warming may be due to short-term warming treatment, experiment warming-induced soil drying, and lower warming magnitude. In contrast, the response of soil inorganic N to experimental warming differed among the three elevations, which may be attributed to different response trends of dissolved organic N and microbial biomass and different initial soil inorganic N.

## Figures and Tables

**Table 1:** Repeated-measures ANOVA (*F* values) for the main and interactive effects of experimental warming (W) and sampling date (D) on soil organic C (SOC), total N (TN), dissolved organic C (DOC), N (DON), ammonium N (NH_4_^+^-N), nitrate N (NO_3_^−^-N), the ratio of NH_4_^+^-N to NO_3_^−^-N (NH_4_^+^-N/NO_3_^−^-N), and soil inorganic N (N_min_, i.e., sum of NH_4_^+^-N and NO_3_^−^-N) in an alpine meadow on the Tibetan Plateau at three elevations (*n* = 4).

Elevation	Model	SOC	TN	DOC	DON	DOC/DON	NO_3_^−^-N	NH_4_^+^-N	NH_4_^+^-N/NO_3_^−^-N	N_min_
4313 m	W	0.02	1.58	0.00	0.23	0.70	39.02**	4.22	1.38	26.87**
	D	0.31	0.26	16.70***	3.66	2.68	55.47***	10.98**	10.04**	28.71***
	W × D	1.04	6.87*	4.13*	2.51	1.40	6.32*	3.44	0.49	5.91*

4513 m	W	1.43	0.03	4.07	5.33	4.52	9.90*	6.45*	3.01	10.89*
	D	2.62	0.23	94.06***	0.99	5.23	57.26***	31.90***	13.69***	51.19***
	W × D	0.41	3.35	2.32	6.36*	6.15*	11.70**	0.63	8.10**	4.39*

4693 m	W	0.40	2.61	0.07	0.09	0.04	0.26	0.00	0.19	0.14
	D	1.96	0.12	0.67	7.80**	0.81	20.22***	21.83***	3.36	29.26***
	W × D	0.27	3.89	0.33	3.84	0.34	0.12	2.66	0.88	1.47

*, **, and *** indicate *P* < 0.05, *P* < 0.01, and *P* < 0.001, respectively, while no asterisk indicates not significant.

**Table 2:** Single factor linear regressions between soil properties (soil organic C, SOC; total N, TN; dissolved organic C, DOC; dissolved organic N, DON; nitrate N, NO_3_^−^-N; ammonium N, NH_4_^+^-N; the ratio of NH_4_^+^-N to NO_3_^−^-N, NH_4_^+^-N/NO_3_^−^-N; soil inorganic N, N_min_) and soil temperature (*T*_*s*_), soil microbial biomass C (MBC), and N (MBN) showing regression parameters (slope, constant, *R* ^2^, and *P*). MBC and MBN data were obtained from Fu et al. [20].

Independent variable	Regression parameters	SOC	TN	DOC	DON	NO_3_^−^-N	NH_4_^+^-N	NH_4_^+^-N/NO_3_^−^-N	N_min_
*T*_*s*_	Slope	−5.29	−0.32	−7.90	−0.53	−0.03	−1.71	−0.43	−1.74
	Constant	100.76	6.81	195.37	13.28	5.73	31.38	7.66	37.11
	*R* ^2^	0.63	0.64	0.38	0.21	0.001	0.31	0.41	0.21
	*P*	<0.001	<0.001	<0.01	0.057	0.93	<0.05	<0.01	0.056

MBC	Slope	0.05	0.003	0.10	0.01	0.01	0.03	0.004	0.03
	Constant	6.46	1.24	46.29	2.89	3.34	−3.20	0.04	0.14
	*R* ^2^	0.76	0.66	0.70	0.51	0.13	0.92	0.47	0.82
	*P*	<0.001	<0.001	<0.001	<0.001	0.139	<0.001	<0.01	<0.001

MBN	Slope	0.28	0.01	0.68	0.05	0.06	0.17	0.02	0.23
	Constant	10.56	1.58	43.22	2.93	1.60	−3.28	0.41	−1.69
	*R* ^2^	0.43	0.30	0.68	0.42	0.39	0.79	0.25	0.88
	*P*	<0.01	<0.05	<0.001	<0.01	<0.01	<0.001	<0.05	<0.001

**Table 3:** Multiple stepwise regression analyses between soil properties and environmental variables (soil temperature, *T*_*s*_; soil moisture, SM) and soil microbial biomass (microbial biomass C, MBC; microbial biomass N, MBN) in an alpine meadow on the Tibetan Plateau. MBC and MBN data were obtained from Fu et al. [20].

Soil properties	Factors	Coefficients	*R* ^2^	*P*
SOC	Constant	49.31		0.003
	MBC	0.04	0.76	<0.001
	*T*_*s*_	−2.69	0.10	0.006

TN	Constant	4.28		0.001
	MBC	0.002	0.66	0.004
	*T*_*s*_	−0.19	0.14	0.006

DOC	Constant	46.29		<0.001
	MBC	0.10	0.70	<0.001

DON	Constant	2.89		0.005
	MBC	0.01	0.51	0.001

NH_4_^+^-N	Constant	−4.57		<0.001
	MBC	0.02	0.92	<0.001
	SM	22.39	0.05	<0.001

NO_3_^−^-N	Constant	1.60		0.22
	MBN	0.06	0.39	0.005

N_min_	Constant	−2.24		0.085
	MBN	0.15	0.88	<0.001
	MBC	0.01	0.05	0.005

NH_4_^+^-N/NO_3_^−^-N	Constant	0.95		0.036
	MBC	0.004	0.47	0.002
